# A New Health Law in Michigan

**Published:** 1889-11

**Authors:** 


### A NEW HEALTH LAW IN MICHIGAN.

### EVERY CASE OF TYPHOID FEVER SHOULD BE REPORTED TO THE HEALTH OFFICER.

Typhoid fever is a disease that the Michigan State Board of Health has declared to be "dangerous to the public health," and as such it comes under the law requiring physicians to report to the health officials. Any physician who shall neglect to immediately give such notice, "shall forfeit for each such offense a sum not less than fifty nor more than $100." *After October 1st, any householder* who shall refuse or wilfully neglect immediately to give such notice, shall be deemed guilty of a misdemeanor, and is liable to a fine of $100, or in default of payment thereof, may be punished by imprisonment in the county jail, not exceeding ninety days.

It seems important that the people generally shall understand this new law, which applies to scarlet fever, diphtheria, small-pox, and all such dangerous diseases, as well as to typhoid fever; but at this time of the year typhoid fever is usually most prevalent, and it is especially dangerous in times of drouth, therefore the safety of the people may now be greatly promoted by having every case of typhoid fever reported to the Health Officer, who is, by law, (Section 1, Act 137, Laws of 1883,) required to promptly attend to the restriction of every such disease. A new law, which takes effect October 1st, makes it a misdemeanor, punishable by fine or imprisonment, for the health officer knowingly to violate that section of the law, or for any person knowingly to violate the orders of the Health Officer made in accordance with that section. But the actual penalties which are incurred by the violation of these laws are the *death penalties* to many people, about 1,000 being lost in Michigan in each year from typhoid fever. The saving of a large portion of these lives is the real reason for the effort, in which it is hoped all persons will join, for the restriction of typhoid fever, and other dangerous diseases.

The attention of the State Board of Health of New York is invited to the wise provisions of this Michigan statute. It would be well to take similar action in this great commonwealth, where so many lives are annually lost from typhoid fever, that might be saved through timely prophylaxis.

